# Complete mitochondrial genome of *Holothuria leucospilata* (Holothuroidea, Holothuriidae) and phylogenetic analysis

**DOI:** 10.1080/23802359.2019.1644226

**Published:** 2019-07-25

**Authors:** Qiuhua Yang, Qi Lin, Jianshao Wu, Ngoc Tuan Tran, Ruifang Huang, Zaiqiao Sun, Zhihuang Zhu, Zhen Lu, Shengkang Li, Chen Zhou

**Affiliations:** a Key Laboratory of Cultivation and High-Value Utilization of Marine Organisms in Fujian Province, Fisheries Research Institute of Fujian, Xiamen, China;; b Guangdong Provincial Key Laboratory of Marine Biology, Marine Biology Institute, Shantou University, Shantou, China

**Keywords:** *Holothuria leucospilata*, mitochondrial genome, phylogenetic analysis

## Abstract

The complete *Holothuria leucospilata* mitochondrial genome was determined and analyzed in this work. It had a circular mapping molecular with a total length of 15,904 bp and contained 13 protein-coding genes, 2 rRNA genes, 22 tRNA genes, and 1 putative control region. Phylogenetic analysis showed that *H. leucospilata* clustered together with *Holothuria scabra* and *Holothuria forskali*. The complete mitochondrial genome provided in this work would be used for elucidation of Holothuroidea conservation genetics and evolutionary relationships.

*Holothuria leucospilata* is one of the broadest distributions of holothurians, which is found in most tropical localities of the Indian Ocean, western central Pacific, and Asian areas (Cherbonnier and Féral 1984). Genetic methods, such as the complete mitochondrial genome, have a great potential to both resolve disputed taxonomic issues and to infer phylogenetic relationships among holothurians (Shen et al. 2009; Perseke et al. 2010; Purcell 2010; Mu et al. 2018).

In this work, we reported and characterized the complete *H. leucospilata* mitogenome (MK940237). One *H. leucospilata* individual (specimen number: 2018091039) was collected from Changjiang, Hainan Province of China (19°26′51″N, 108°51′56″E) and stored at −80 °C in the Culture Collection of Sea cucumber at the Fisheries Research Institute of Fujian of China for DNA isolation.

The complete *H. leucospilata* mitogenome is a circular DNA molecule with a length of 15,904 bp. The gene arrangement is identical to the echinoderm ground pattern (Scouras et al. 2004; Fan et al. 2012), including 13 protein-coding genes (PCGs), 2 rRNA genes, 22 tRNA genes, and a putative control region (PC-region). The overall base composition of its heavy strand is 31.42% (A), 26.13% (T), 25.89% (C), and 16.56% (G) showing a bias toward A + T (57.55%). The 13 PCGs encode 3777 amino acids in total. Leucine (16.39%) is the most frequently used amino acid, while cysteine acid (0.95%) is the least frequently used one. The PC-region is 551 bp in length and locates between the *tRNA-Thr* and *tRNA-Pro* genes with a higher A + T content (58.98%). Apart from the PC-region, there are 16 small intergenic spacers that range from 1 to 18 bp in size, totally 104 bp. In total, six overlapping areas (24 bp) were observed, in which the three notable overlaps (*ATP8* and *ATP6* by 7 bp, *nad4* and *tRNA-His* by 10 bp, and *tRNA-Pro* and *tRNA-Gln* by 4 bp) were similar to other echinoderms (Fan et al. 2011). Twenty-two tRNA genes were identified in the mitogenome of *H. leucospilata*. The *16S* rRNA is 1563 bp in length and locates between *nad2* and *cox1*, while the *12S* rRNA is 830 bp in length and locates between *tRNA-Phe* and *tRNA-Glu*.

A maximum likelihood phylogenetic tree of 12 species in the class Holothuroidea and the two-outgroup species (KC490911 and EU054306) was constructed based on the concatenated amino acid of 13 PCGs in echinoderm (Figure 1). Comparative mitogenome analyses between *H. leucospilata* and other holothurians disclosed that the mitogenome of *H. leucospilata* is highly compacted in its organization. Pairwise genetic p-distances of 13 PCGs between *H. leucospilata* and *H. scabra* (KP257577) vary from 8.68% (*cox1*) to 56.91% (*atp8*) and the genetic distance between them for the concatenated 13 PCGs is 20.08%. This confirmed the findings in a previous study that the genus *Holothuria* (belonging to the order Aspidochirotida) is much older than the genus *Stichopus* (Uthicke et al. 2009; Xia et al. 2016).

**Figure 1.**
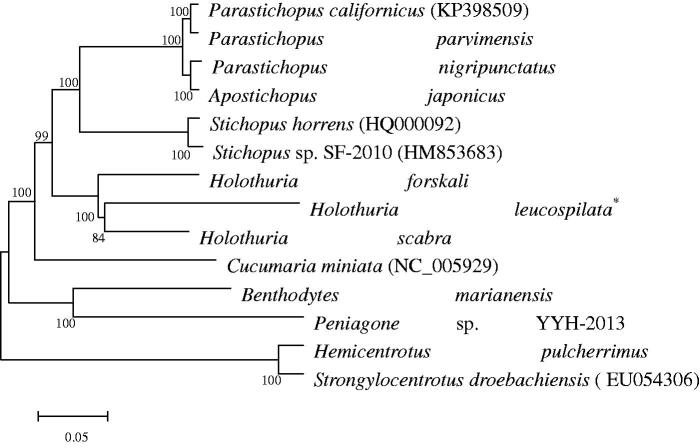
Phylogenetic tree (maximum likelihood) based on the concatenated amino acid of 13 protein-coding genes. The bootstrap values are based on 1000 re-samplings. The number at each node is the bootstrap probability. The number after the species name in the brackets is the GenBank accession number. The asterisks after species names indicate newly determined mitochondrial genomes.
